# COVID-19 and fortuitous discovery of chronic lymphocytic leukemia: biological findings and therapeutic challenges

**DOI:** 10.11604/pamj.2020.36.286.24361

**Published:** 2020-08-17

**Authors:** Boubaker Charra, Ayman Ellouadghiri, Abdellah Magramane, Touda Kebbou, Kenza Damaan, Abdeljabbar Maghfour, Kamal Seddiki, Hanane Ezzouine

**Affiliations:** 1 Department of Anesthesiology and Intensive Care, Ibn Rochd University Hospital of Casablanca, Hassan II University, Casablanca, Morocco,; 2 Department of Radiology, Ibn Rochd University Hospital of Casablanca, Hassan II University, Casablanca, Morocco

**Keywords:** COVID-19, SARS-CoV-2, CLL, diagnosis, therapy

## Abstract

With the major spread of SARS-COV-2 around the world, its association with various pathologies has been reported. However, hemopathy has rarely been revealed during a coronavirus infection. The authors of this article aim to emphasize the diagnostic and therapeutic challenges faced while treating COVID/hemopathy patients.

## Introduction

Since December 2019, at the beginning of the outbreak in Wuhan China, SARS-COV-2 has been a one the biggest challenges public health has faced. As of today, over 7.8 million people have been infected and we deplore more than 450,000 deaths. Immunodeficient individuals are considered at high risk of developing severe forms of COVID-19. The association COVID-19 haemopathy is more frequent nowadays and is considered as a real challenge for physicians. The authors report the case of fortuitous discovery of chronic lymphocytic leukemia in a COVID-19 patient, emphasizing the difficulties of therapeutic care.

## Patient and observation

**Figure 1:**
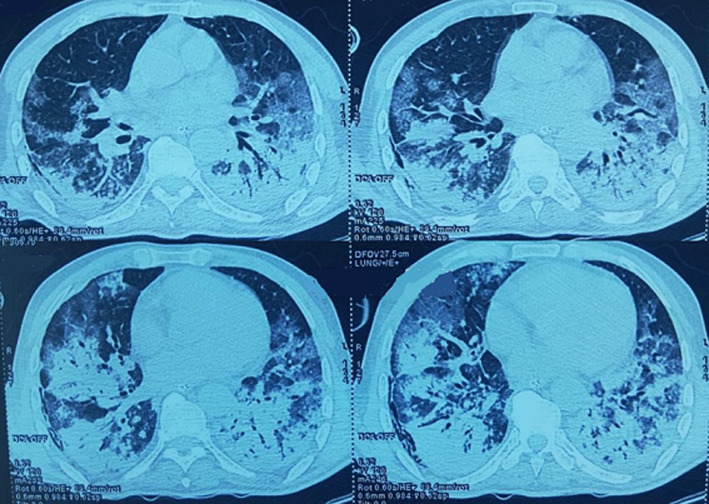
chest CT scan showing bilateral ground glass opacities with consolidation

A 76-year-old male, with a history of colon cancer treated in 2013 (surgery), admitted in the intensive care unit for SARS-CoV-2 pneumonia. The patient has reported a stay in an endemic country 21 days before the admission. The main symptoms were dry cough, 40°C fever, multiples adenopathies and diarrhea. The patient´s state worsened and complicated by respiratory distress leading to a transfer to intensive care unit. A PCR test was conducted and came back positive. Chest CT scan showed bilateral ground glass opacities with consolidation (Figure 1). During his admission, the patient was conscious, GCS at 15/15, polypneic at 30 cycles/min, SpO2 of 85% at breathing room air with intercostal retraction, stable hemodynamics: BP 140/75mmHg, HR at 90bpm and 39°C fever. EKG showed normal sinus rhythm, fixed duration of PR interval, QTc at 475. Transthoracic echocardiography was normal. Arterial blood gas showed pH at 7.46, PaCO2 at 48mmHg, HCO3- at 26mmol/L, PaO2 at 131mmHg, PaO2/FiO2 ratio at 257.

Lab tests conducted at D1 of admission, revealed elevated WBC at 140020el/mm^3^and lymphocytes 129660el/mm^3^(vs 154000el/mm^3^at D7), low hemoglobin at 8.9g/dL, platelets at 464000el/mm^3^, PT at 64%, fibrinogen at 6.77g/L, CRP at 130mg/L, BNP at 249pcg/L, PCT at 0.017μg/L, LDH at 331UI/L, ferritin at 563μg/L, troponin at 8.2ng/L. Blood smear showed small lymphocytes with rounded nucleus and reduced cytoplasm. Immunophenotyping of peripheral blood confirmed the presence of B-cell population; expressing one light chain immunoglobulin. Our therapeutic care was based on four daily noninvasive ventilation (NIV) sessions, hydroxychloroquine (200mg, twice a day), azythromycin (500mg per day), ceftriaxon (2g per day) and moxifloxacin (400mg, twice a day). The patient also received preventive dose of human immunoglobulins (0.5g/kg single dose), anti-coagulation, PPI, vitamin C, vitamin D and zinc. The patient´s respiratory status worsened leading to intubation and mechanical ventilation. Severe ARDS followed and 10 days after his admission, the patient expired.

**Ethical approval:** informed consent was obtained from the patient´s family for publication of this case.

## Discussion

Chronic lymphocytic leukemia (CLL) is the most frequent leukemia during adulthood. Eighty percent of the patients are asymptomatic at the moment of the diagnosis and 30% will never receive a treatment [1]. CLL is characterized with a clonal proliferation and accumulation of mature B cells, usually CD5 positive, in the bloodstream, bone marrow, lymph nodes and spleen [2]. The diagnosis of CLL is based on the presence of at least 5000 B-cell lymphocytes/μL in peripheral blood stream for at least three months. Clonality of circulating B cells must be confirmed by flow cytometry. The abnormal cells found at a blood smear test are typically mature small lymphocytes with a cytoplasmic border and dense nucleus deprived of nucleoli with partially aggregated chromatin [2, 3]. Treatment is based on immunochemotherapy (Table 1) for 90% of the patients with no genetic abnormalities associated with chemo resistance [4, 5]. The use of new therapies cibling different pathways is more frequent nowadays and could become an alternative to chemotherapy in the future. The association SARS-COV-2/CLL is a real challenge for physicians as they both have their own specific therapies.

**Table 1:** US food and drug administration-approved drugs for the treatment of chronic lymphocytic leukemia

Frontline Setting	Relapsed Setting
Alkylating agents: bendamustine; chlorambucil; cyclophosphamide	Ibrutinib
Purine analogs: fludarabine; pentostatin	Phosphatidylinositol-3-kinase inhibitor idelalisib + rituximab duvelisib
Anti-CD20 monoclonal: antibodies; obinutuzumab; ofatumumab; rituximab	BCL-2 inhibitor (venetoclax) +/− rituximab
Bruton tyrosine kinase: inhibitor; ibrutunib	Anti-CD20 monoclonal antibodies: obinutuzumab; ofatumumab; rituximab
	Cellular therapies: allogenic hematopoietic stem cell transplantation

Abbreviations: +/−, with or without; anti-CD20, anti-B-lymphocyte antigen cluster of differentiation; 20 BCL-2, B-cell lymph

Individuals with a compromised immunity can have longer incubation period of the virus [6]. Our patient had an incubation period of 21 days. SARS-COV-2 induced lymphopenia is correlated with severe forms of the disease in general population [7, 8]. In our case, we have noted a significant increase of lymphocyte population after a week. This increase has been previously reported by a British study [9], however the mechanisms are still unknown. The main therapeutic challenge in this case is due to the lack of consensus regarding COVID-19 and hemopathies. The treatment should therefore be personalized. The association of intravenous human immunoglobulins is highly recommended in patients presenting repeated infections, in order to boost their immunity [10]. However, the administration of chemotherapy is still controversed, as it can worsen SARS-COV-2 immunodeficiency, lead to cardiotoxicity and aggravate the prognosis. Chemotherapy should be avoided for CLL patients presenting COVID-19 to restrain treatment-related immunodeficiency and prevent drug interactions [10].

## Conclusion

Clinical and biological symptoms of COVID-19 can be concealed due to its coexistence with malignant hemopathies such as chronic lymphocytic leukemia. Treatment should be personalized according to the patient´s immune status and comorbidities.
